# The Current Landscape of Remote Patient Monitoring Regarding Diabetes Mellitus and Hypoglycemia: Protocol for a Scoping Review

**DOI:** 10.2196/88197

**Published:** 2026-04-29

**Authors:** Mathew J Stephen, Baylor Akhavan, Melinda B Chu, Kavita Batra, Roberto Sagaribay

**Affiliations:** 1 Department of Biology University of Rochester Rochester, NY United States; 2 Department of Medical Education Kirk Kerkorian School of Medicine University of Nevada, Las Vegas Las Vegas, NV United States; 3 Auriva Health, Inc Blue Ash, OH United States; 4 Office of Research Kirk Kerkorian School of Medicine University of Nevada, Las Vegas Las Vegas, NV United States

**Keywords:** remote patient monitoring, diabetes management, hypoglycemia prevention, continuous glucose monitoring, digital health technologies

## Abstract

**Background:**

Diabetes mellitus is characterized by impaired glucose regulation, predisposing patients to both hyperglycemia and hypoglycemia. Hypoglycemia, particularly frequent in insulin-treated individuals, remains a serious but underrecognized complication. Remote patient monitoring (RPM) technologies, such as continuous glucose monitors, smartphone apps, and hybrid closed-loop systems with remote monitoring capabilities, have emerged as promising tools to improve glycemic control and prevent hypoglycemia in nonclinical settings. Evidence examining the use of RPM technologies has expanded rapidly; however, the scope, characteristics, and reported outcomes of these interventions remain fragmented across modalities and settings.

**Objective:**

This scoping review aims to systematically map and describe the extent, range, and characteristics of published evidence on RPM interventions for glycemic management among adults with type 1 and type 2 diabetes in nonclinical settings.

**Methods:**

The review will follow the Joanna Briggs Institute scoping review methodology and adhere to the PRISMA-ScR (Preferred Reporting Items for Systematic Reviews and Meta-Analyses Extension for Scoping Reviews) reporting guidelines. The population, concept, and context framework defines the population as adults with type 1 or type 2 diabetes who experience or are at risk for hypoglycemia; the concept as RPM techniques (continuous glucose monitors, hybrid closed-loop systems with remote monitoring capabilities, telemedicine, and smartphone apps); and the context as nonclinical environments. The PubMed, Embase, and Scopus databases will be searched, supplemented by gray literature. Eligible studies will include clinical trials, observational studies, and cohort studies; reviews, case studies, and non-English articles will be excluded. Two independent reviewers will conduct screening, data extraction, and summarization. Findings will be synthesized descriptively in tabular and narrative formats.

**Results:**

At the time of submission, the protocol has been registered, and the formal search strategy is being finalized. As of March 2026, the formal database search has been completed, and screening of studies is scheduled to begin in April 2026.

**Conclusions:**

This protocol outlines a structured approach to mapping the current landscape of RPM interventions for glycemic management in nonclinical settings. The completed review will synthesize reported intervention characteristics and outcomes to clarify existing evidence and identify areas for future investigation.

**Trial Registration:**

OSF Registries 10.17605/OSF.IO/XNBWF; https://osf.io/xnbwf/overview

**International Registered Report Identifier (IRRID):**

PRR1-10.2196/88197

## Introduction

### Background

Glucose regulation in humans depends on the antagonistic actions of insulin and glucagon, hormones secreted by pancreatic beta and alpha cells that work together to maintain glucose homeostasis [1]. In diabetes mellitus, this balance is disrupted: type 1 arises from autoimmune destruction of beta cells leading to absolute insulin deficiency, while type 2 involves progressive insulin resistance [2]. Both conditions confer risk for hypoglycemia and hyperglycemia; however, hypoglycemia often results from excessive insulin dosing, missed meals, or physical activity, whereas hyperglycemia stems from inadequate insulin or excessive glucagon activity [3,4]. Hypoglycemia is a serious but underrecognized complication, contributing to physical, cognitive, and psychological harm, especially when repeated episodes impair awareness and counterregulation [5,6]. It is also a major driver of emergency care use, reduced treatment adherence, and diminished quality of life, making its prevention a critical public health and clinical priority.

Maintaining glucose within the recommended preprandial range of 80 to 130 mg/dL is critical to avoid these complications [2]. These management challenges underscore the growing role of remote patient monitoring (RPM), which enables continuous surveillance of glucose fluctuations and more timely interventions [4]. By shifting care from reactive to proactive management, RPM technologies provide opportunities not only to reduce acute episodes of hypoglycemia but also to lessen long-term complications and health care costs [7].

The importance of RPM is underscored by the high prevalence of hypoglycemia in both type 1 and type 2 diabetes. In a patient-reported study, hypoglycemic events were reported in 97.4% of patients with type 1 diabetes and in 95.3% of patients with type 2 diabetes over just a 4-week period [8]. Thus, hypoglycemia remains a near-universal complication that requires innovative solutions to address.

RPM offers substantial value in the management of diabetes by enabling continuous, real-time tracking of blood glucose levels and facilitating timely clinical interventions. Tools such as continuous glucose monitors, smartphone-based apps, and hybrid closed-loop systems with remote monitoring capabilities allow for greater patient autonomy, improved treatment adherence, and reduced reliance on in-person visits [9-11]. Evidence has examined the association between RPM interventions and various glycemic outcomes, including hemoglobin A_1c_ (HbA_1c_) levels, time in range, and reported hypoglycemic events [10,11]. However, findings vary across technologies, study designs, and care settings, and the literature remains dispersed across multiple intervention types.

Existing scoping and systematic reviews examining diabetes management through remote monitoring and digital interventions exhibit several important limitations in scope and focus. Many reviews emphasize narrow aspects of diabetes care—such as health coaching [12], mobile app–based self-management [13], or inpatient continuous glucose monitoring (CGM) implementation [14]—rather than providing a comprehensive synthesis of RPM modalities for glycemic control across settings. Others are restricted to specific populations, such as Arabs with diabetes [15], or discuss chronic disease management broadly without a targeted focus on diabetes [16,17]. Several reviews address artificial intelligence (AI) integration in clinical workflows [15,17] but lack depth regarding traditional CGM and home-based monitoring technologies, particularly their application in outpatient diabetes care.

The proposed scoping review will address these limitations by systematically mapping the breadth of RPM interventions used for both type 1 and type 2 diabetes management in outpatient and home settings. Unlike prior reviews, it will integrate traditional and AI-enhanced monitoring technologies, encompass a range of RPM modalities beyond mobile apps, and include diverse patient populations irrespective of demographic or geographic constraints. By synthesizing the available evidence on implementation contexts, intervention types, and clinical and behavioral outcomes, this scoping review will provide a comprehensive overview of the current landscape of remote monitoring in diabetes care and identify critical gaps to guide future empirical research and policy development.

Given the high prevalence of hypoglycemia among individuals with type 1 and type 2 diabetes, there is a growing need to understand how RPM technologies are being used in nonclinical settings to prevent hypoglycemic events and improve overall glycemic control. This scoping review is designed to systematically map the range of RPM modalities evaluated in nonclinical settings and to describe how outcomes, including hypoglycemia-related measures, have been reported. A preliminary search of Medical Subject Headings (MeSH) terms (diabetes, hypoglycemia, and RPM) in the PubMed database identified 8 articles, which were evaluated to determine whether any existing reviews address our investigation.

### Study Objectives

The objective of this scoping review is to systematically map and describe the extent, range, and characteristics of published evidence on RPM interventions for glycemic management among adults with type 1 and type 2 diabetes in nonclinical settings.

This review aims to provide a structured overview of the types of RPM interventions studied, the populations included, the care contexts examined, and the outcomes reported in the existing literature.

### Review Question

How are RPM interventions being implemented and evaluated for glycemic management among adults with type 1 and type 2 diabetes in nonclinical settings? To address this overarching question, the review will consider the following subquestions:

- What types of RPM interventions (eg, CGM systems, hybrid closed-loop systems with remote data transmission, and structured telemonitoring platforms) have been studied in nonclinical settings?
- Which populations have been included in these studies (eg, diabetes type, age groups, and clinical risk profiles)?
- What glycemic and hypoglycemia-related outcomes have been reported (eg, frequency of hypoglycemia, time in range, and HbA _1c_)?
- How have implementation characteristics, usability considerations, accessibility factors, and care settings been described?
- How are emerging technologies, including digital health platforms and AI-enabled systems, represented within the current evidence base?

A preliminary search conducted on August 10, 2025, found no ongoing or published scoping or systematic reviews specifically addressing RPM for type 1 and type 2 diabetes in nonclinical settings with a focus on hypoglycemia prevention.

## Methods

### Protocol Registration

Given that this study does not involve human subjects, institutional ethics review will not be required. The protocol was registered on August 6, 2025, through the Open Science Framework [18], a database for review registrations to promote transparency, prevent duplication, and reduce reporting bias [19].

### Inclusion and Exclusion Criteria

#### Overview

The proposed scoping review will follow the population, concept, context (PCC) framework for structuring review questions, identifying literature, and developing inclusion criteria (Table 1).

**Table 1:** Population, concept, and context (PCC) framework.

PCC	Description
Population	Adults with type 1 or type 2 diabetes who experience or are at risk for hypoglycemia
Concept	RPM^a^ techniques involving remote physiological monitoring (eg, continuous glucose monitoring, hybrid closed-loop systems with remote data transmission, and structured telemonitoring platforms) Telemedicine-only interventions without remote physiological data monitoring will be excluded
Context	Nonclinical environments (home settings)

^a^RPM: remote patient monitoring.

#### Population

The population includes adults with type 1 or type 2 diabetes who experience or are at risk for hypoglycemia. Studies focusing exclusively on gestational diabetes, pediatric populations, or nondiabetic hypoglycemia will be excluded.

#### Concept

The concept of interest is RPM interventions involving remote physiological glucose monitoring through digital data capture and transmission. Eligible interventions include CGM systems, hybrid closed-loop systems with remote data-sharing capabilities, and structured telemonitoring platforms that enable remote clinician review of glucose data. Interventions consisting solely of telemedicine consultations (eg, video or telephone visits without remote physiological data transmission) will be excluded to maintain conceptual consistency with the operational definition of RPM used in this review.

#### Context

The context includes nonclinical environments, such as home and outpatient settings. Studies conducted exclusively in inpatient hospital settings will be excluded.

#### Study Designs

Eligible studies will include primary empirical research designs such as randomized controlled trials, cohort studies, cross-sectional studies, and other observational designs. Reviews (including systematic and scoping reviews), narrative reviews, editorials, conference abstracts, posters, case reports, and case series will be excluded, as the objective of this scoping review is to map and characterize primary research evidence. However, the reference lists of relevant reviews will be screened to identify additional eligible primary studies. Only studies published in English will be included due to feasibility and resource limitations for translation. This restriction is acknowledged as a potential source of language bias. Consistent with the Joanna Briggs Institute (JBI) methodology for scoping reviews, no restrictions will be placed on publication year to capture the evolution of RPM technologies over time.

### Informational Sources and Search Strategy

This scoping review will follow the methodology outlined by the JBI for conducting scoping reviews [20].

#### Preliminary Search for Planned or Ongoing Studies

A preliminary search was conducted on August 10, 2025, in the following databases: PROSPERO, PubMed (with a systematic review filter), the Cochrane Database of Systematic Reviews, and JBI Evidence.

In PROSPERO, 20 protocols were identified with partial topical overlap. Most examined RPM in diabetes care broadly, hypoglycemia in specific ethnic subgroups, or CGM use without focusing on nonclinical environments. None addressed the combined scope of this review—*hypoglycemia prevention in both type 1 and type 2 diabetes using RPM tools in home and outpatient settings*.

In the MeSH analysis, 8 PubMed-indexed articles were identified with relevant MeSH terms (eg, “Blood Glucose Self-Monitoring/methods,” “Artificial Intelligence*,” and “Algorithms”). These covered topics such as mobile health app–assisted self-care, AI in diabetes management, and CGM use; however, either they were narrative in nature or they addressed only a subset of our inclusion criteria.

Given the absence of a review meeting our specific PCC framework, this study addresses a clear gap in the literature and will complement existing work by focusing on the intersection of nonclinical RPM, both major types of diabetes, and hypoglycemia prevention.

#### Search Strategy

On the basis of the preliminary findings, keywords and index terms will be compiled and refined. A comprehensive search strategy will then be developed and adapted for each selected database. The full search will be conducted in the PubMed, Embase, and Scopus databases using all identified search terms and database-specific indexing. Third, the reference lists of all included reports and articles will be screened to identify additional relevant sources. Authors of primary studies may be contacted for clarification or to obtain missing information where necessary. The complete search strategies for each database are provided in Multimedia Appendix 1.

In addition to database searches, gray literature will be explored through structured sources including ClinicalTrials.gov, the World Health Organization International Clinical Trials Registry Platform, and the Open Science Framework registries. Google Scholar will be used as a supplementary source to identify additional potentially relevant materials. Due to the reproducibility limitations and decreasing precision of relevance-ranked results beyond early records, screening will be limited to the first 100 results sorted by relevance.

#### Peer Review of Electronic Search Strategies Review

A formal Peer Review of Electronic Search Strategies (PRESS) review is planned for November 2025 by the medical education librarian at the University of Rochester [21].

### Study Selection and Screening

Following the literature search, the reference management software RefWorks (Clarivate) will be used to organize citations, remove duplicates, and exclude clearly irrelevant records such as narrative reviews. Title and abstract screening will be conducted using Rayyan (Rayyan Systems Inc**)** by 2 independent reviewers in strict alignment with the predefined inclusion criteria. Full-text articles for all studies deemed potentially eligible will then be retrieved, and their citation data will be reimported into the reference manager to create a separate, curated library.

This finalized collection will be uploaded into Rayyan for full-text screening. At this stage, each article will be evaluated to confirm eligibility based on the inclusion criteria. Reasons for exclusion will be recorded and reported in the final review. Any discrepancies between reviewers will be resolved through discussion or by consulting a third reviewer. The final list of included studies will be documented with complete citations and summarized in a PRISMA-ScR (Preferred Reporting Items for Systematic Reviews and Meta-Analyses Extension for Scoping Reviews) flow diagram to illustrate the study selection process [22].

### Data Extraction

Two independent reviewers will extract data from full-text articles included in the scoping review. The extraction form may be modified as needed, with all changes documented in the final review. Discrepancies will be resolved by a third reviewer. A draft data extraction form will be developed and pilot-tested on a subset of included studies to ensure clarity, completeness, and consistency. Any modifications to the form will be documented prior to full data extraction. Consistent with the JBI methodology for scoping reviews, a formal critical appraisal or risk-of-bias assessment will not be conducted. The objective of this review is to map the breadth and characteristics of the available evidence rather than to evaluate intervention effectiveness.

### Data Analysis and Presentation

Data from included studies will be analyzed descriptively to map the scope and characteristics of the literature on RPM for glycemic control among patients with type 1 and type 2 diabetes at risk for hypoglycemia in nonclinical settings. Extracted data will include study design; location; diabetes type; RPM tools used (eg, continuous glucose monitors, AI-driven systems, smartphones, and smartwatches); outcomes measured; study populations; care settings; and ethical oversight. Results will be presented through summary tables and charts, supplemented by a narrative synthesis to identify key themes, trends, and gaps in the literature. Only interventions incorporating remote glucose data capture and monitoring will be included to maintain conceptual consistency with the definition of RPM. Studies involving gestational diabetes, nondigital interventions, or inpatient clinical settings will be excluded and documented accordingly.

### Ethical Considerations

Ethics approval of this study was not required as this scoping review encompasses previously published material.

## Results

The scoping review was conducted from August 2025 to March 2026. Idea conception and protocol registration occurred between August and September 2025, followed by a preliminary search and PRESS review in October 2025. The formal database search was completed in November 2025. As of March 2026, screening of identified studies has not yet begun and is scheduled to occur in April 2026, followed by data extraction and synthesis.

## Discussion

### Anticipated Findings

As this manuscript presents a scoping review protocol, this section focuses on the rationale and anticipated methodological contribution of the review rather than the interpretation of findings. RPM technologies have expanded considerably in diabetes care, particularly in outpatient and home settings. A growing body of literature has evaluated CGM systems, hybrid closed-loop systems, and related telemonitoring platforms in adults with type 1 and type 2 diabetes [22-24]. As intervention modalities diversify including wearable sensors, hybrid closed-loop systems with remote monitoring capabilities, and mobile health apps, variability in how RPM is defined, implemented, and evaluated across studies is increasing. A structured synthesis of this evidence is therefore necessary to clarify the scope, characteristics, and contexts in which these interventions have been examined.

This scoping review will systematically map the types of RPM interventions studied in adults with type 1 and type 2 diabetes in nonclinical environments. By describing intervention components, study populations, care settings, and reported outcomes, including measures related to hypoglycemia such as frequency, severity, and time in range, the review will provide conceptual clarity regarding how RPM is operationalized across the literature. Particular attention will also be given to how usability, patient experience, and access-related factors have been reported in existing studies [23].

Rather than evaluating intervention effectiveness or drawing conclusions about clinical impact, this review is designed to identify patterns, research gaps, and areas of concentration within the current body of evidence. The resulting evidence map may support future research prioritization, methodological standardization in outcome reporting, and more consistent definitions of RPM in diabetes care research.

### Strengths and Limitations

A major strength of this study is its timeliness, as RPM technologies have expanded rapidly in the wake of the COVID-19 pandemic. By focusing specifically on hypoglycemia prevention in both type 1 and type 2 diabetes across nonclinical settings, this review addresses a critical gap not covered by prior scoping or systematic reviews. Another strength is methodological rigor: the review will follow the JBI framework and PRISMA-ScR guidelines, with independent screening and data extraction by 2 reviewers to reduce bias. Including both peer-reviewed and gray literature also increases the likelihood of capturing emerging and real-world practices not yet widely represented in academic publications.

Despite these strengths, several limitations should be acknowledged. First, heterogeneity in study designs, populations, and reported outcomes may limit direct comparisons. Second, the exclusion of non-English studies could bias the evidence toward higher-income, English-speaking countries, underrepresenting data from other regions. Third, as with any evidence synthesis, publication bias is a concern, since studies with positive or favorable outcomes are more likely to be published, potentially skewing the evidence base. Fourth, because digital health technologies evolve quickly, some findings may become outdated unless regularly updated. Finally, while gray literature will be included, variability in reporting standards and methodological transparency may limit the ability to fully assess study quality.

### Future Directions

This review will be used to identify gaps in outcome standardization, reporting consistency, and implementation evaluation within RPM research. Areas requiring further investigation may include long-term follow-up, comparative effectiveness across modalities, equity-focused implementation strategies, and integration of AI-enabled tools. By mapping these gaps, the review will support the development of more rigorous and targeted future studies.

### Dissemination Plan

A comprehensive dissemination plan will take place, beginning with a publication in a peer-reviewed journal and presentations at academic conferences and annual meetings.

### Conclusions

This scoping review protocol outlines a systematic approach to mapping the existing literature on RPM interventions for glycemic management among adults with type 1 and type 2 diabetes in nonclinical settings. By synthesizing the range of intervention types, populations studied, and reported outcomes, particularly those related to hypoglycemia, the completed review will clarify the current evidence landscape and identify areas requiring further research and methodological refinement.

## Appendix

Multimedia Appendix 1 Complete database-specific search strategies.

## Abbreviations

AI: artificial intelligence
CGM: continuous glucose monitoring
HbA1c: hemoglobin A1c
JBI: Joanna Briggs Institute
MeSH: Medical Subject Headings
PCC: population, concept, and context
PRESS: Peer Review of Electronic Search Strategies
PRISMA-ScR: Preferred Reporting Items for Systematic Reviews and Meta-Analyses Extension for Scoping Reviews
RPM: remote patient monitoring

## Data Availability

Data sharing is not applicable to this paper as no datasets were generated or analyzed during this study.
