# Supplementary material for: The Current Landscape of Remote Patient Monitoring Regarding Diabetes Mellitus and Hypoglycemia: Protocol for a Scoping Review
Source: JMIR Res Protoc. 2026 Apr 29;15:e88197. doi: 10.2196/88197 (PMC13173060; doi:10.2196/88197)
Supplement: Multimedia Appendix 1 [file resprot_v15i1e88197_app1.docx]

**Table S1. Complete Database-Specific Search Strategies**

| **Database** | **Search Strategy** |
| --- | --- |
| **PubMed** | ("Diabetes Mellitus, Type 1"[Mesh] OR "Diabetes Mellitus, Type 2"[Mesh] OR "type 1 diabetes" OR "type 2 diabetes" OR T1DM OR T2DM) AND ("Remote Patient Monitoring"[Mesh] OR "remote patient monitoring" OR telemonitoring OR telemedicine OR "digital health" OR mHealth OR "mobile health" OR "continuous glucose monitoring" OR CGM OR "hybrid closed loop" OR "artificial pancreas" OR "wearable glucose monitor") AND ("Hypoglycemia"[Mesh] OR hypoglycemia OR "low blood glucose" OR "glycemic control" OR HbA1c OR "time in range") |
| **Embase** | ('type 1 diabetes'/exp OR 'type 2 diabetes'/exp OR 'type 1 diabetes' OR 'type 2 diabetes' OR T1DM OR T2DM) AND ('remote patient monitoring'/exp OR 'telemonitoring'/exp OR telemonitoring OR telemedicine OR 'digital health' OR mhealth OR 'continuous glucose monitoring'/exp OR CGM OR 'hybrid closed loop' OR 'artificial pancreas' OR 'wearable glucose monitor') AND ('hypoglycemia'/exp OR hypoglycemia OR 'low blood glucose' OR 'glycemic control' OR hba1c OR 'time in range') |
| **Scopus** | TITLE-ABS-KEY("type 1 diabetes" OR "type 2 diabetes" OR T1DM OR T2DM) AND TITLE-ABS-KEY("remote patient monitoring" OR telemonitoring OR telemedicine OR "digital health" OR mhealth OR "continuous glucose monitoring" OR CGM OR "hybrid closed loop" OR "artificial pancreas" OR "wearable glucose monitor") AND TITLE-ABS-KEY(hypoglycemia OR "low blood glucose" OR "glycemic control" OR HbA1c OR "time in range") |
